# Supplementary material for: Molecular tuning of farnesoid X receptor partial agonism
Source: Nat Commun. 2019 Jul 2;10:2915. doi: 10.1038/s41467-019-10853-2 (PMC6606567; doi:10.1038/s41467-019-10853-2)
Supplement: Supplementary file 1 — Supplementary Information [file 41467_2019_10853_MOESM1_ESM.pdf]

Supplementary Information for

Molecular tuning of farnesoid X receptor partial agonism

D. Merk, S. Sreeramulu et al.

Supplementary Figures

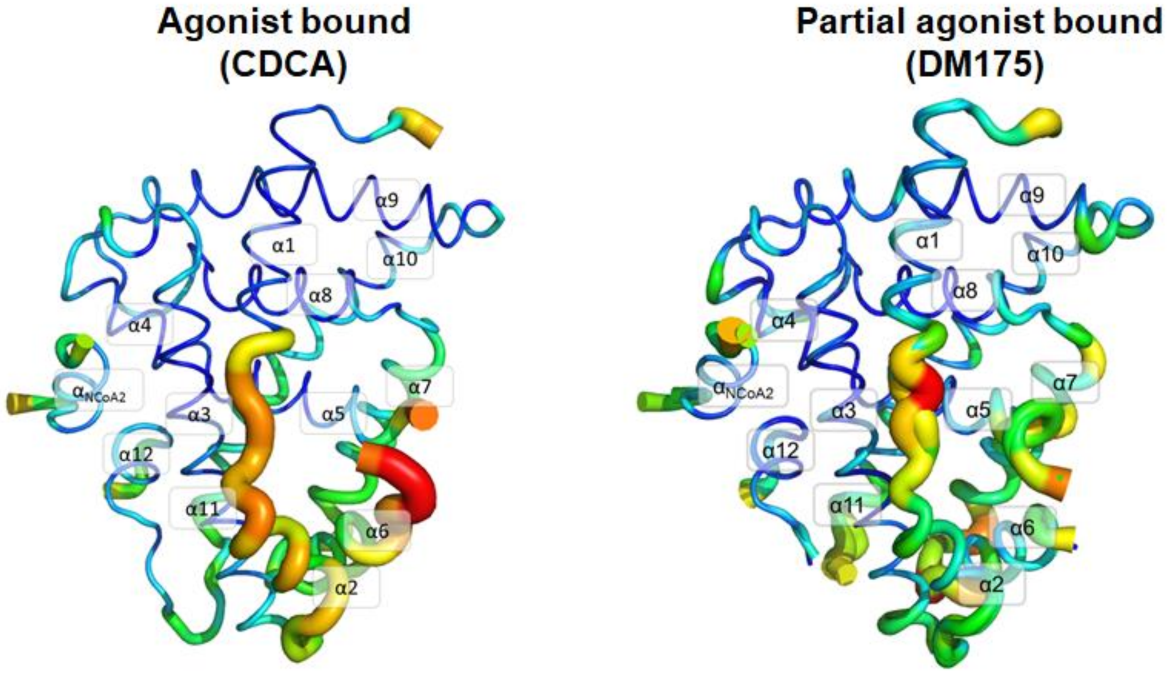

Supplementary Figure 1: Influence of ligand binding on temperature factors. Blue/slim backbone is equal to low temperature/stable; red/large backbone is equal to high temperature/flexible. ω-loop (region between helix-1 and helix-3) is destabilized by the partial agonist compared to the agonist (CDCA).

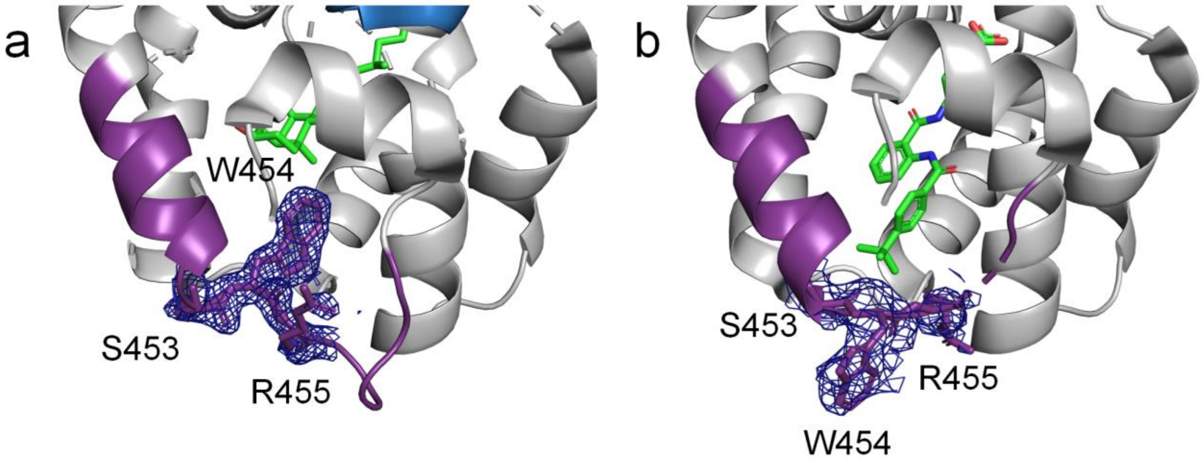

Supplementary Figure 2: Electron density for W454 and neighboring residues in CDCA (a) and partial agonist (1) bound (b) FXR LBD structures. The electron density (2Fc-Fo omit map, contour level 1.0 σ) of W454 can clearly be assigned and shows an outward movement of this residue in the partial agonist bound state.

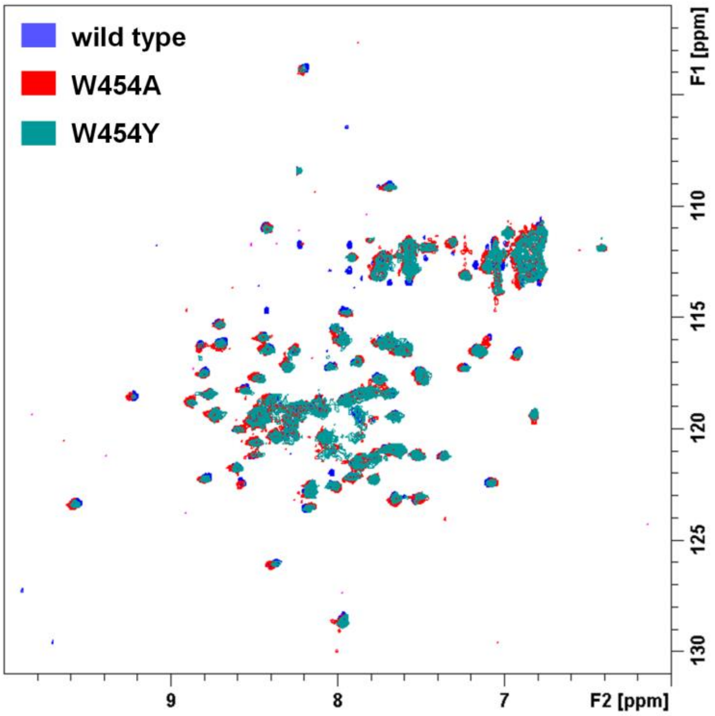

Supplementary Figure 3: $^{1}H/^{15}N$-HSQC spectra of fully labeled ligand binding domain of wild type FXR and mutants W454A and W454Y.

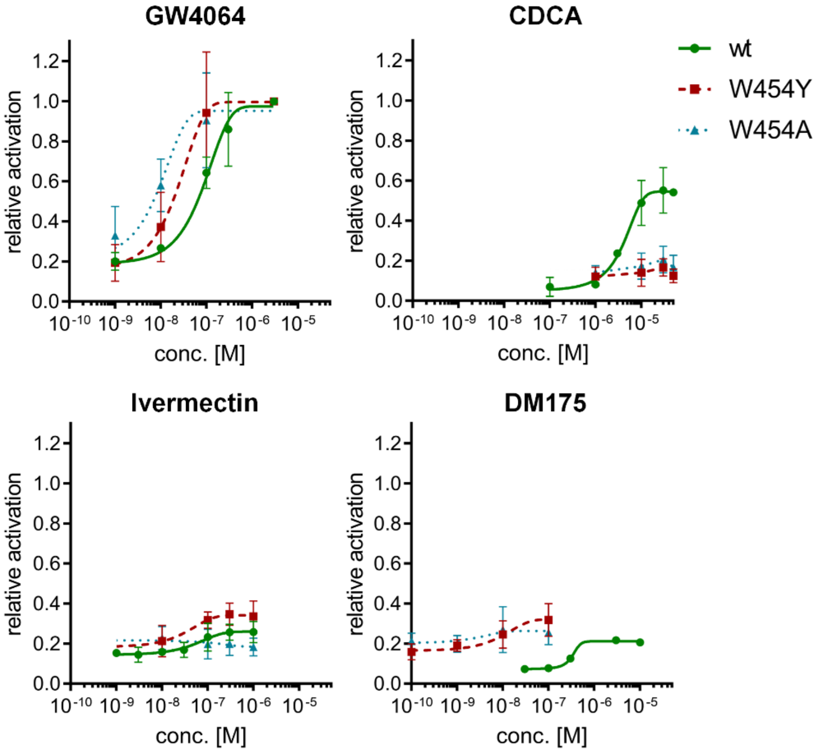

Supplementary Figure 4: Activity of FXR agonists and partial agonists on wild-type FXR and mutants W454Y and W454A. Dose-response curves of GW4064, CDCA, Ivermectin and DM175 (**1**) on wt FXR and the mutants W454Y and W454A in BSEP-based full-length reporter gene assays. Results are the mean±SEM, n=3. Source data are provided as a Source Data file.

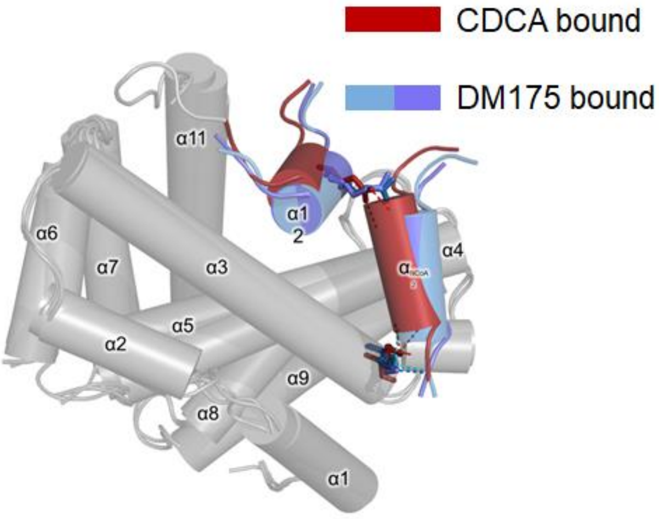

Supplementary Figure 5: Influence of agonist (CDCA) and partial agonist (DM175) binding on the AF-2 helix (α12) and co-activator peptide binding to FXR.

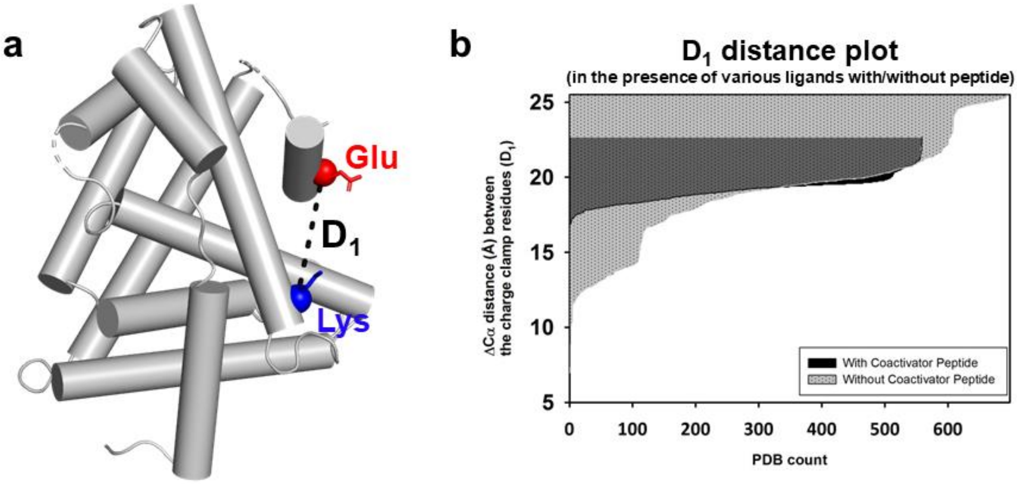

Supplementary Figure 6: Key salt-bridge distance analysis of approx. 1300 NR structures deposited in the PDB. (a) Schematic representation of the common NR structure with Lys (α3) and Glu (α12) forming a dipolar "charge clamp" for co-activator recruitment. D_1 indicates the distance between Cα of both residues. (b) Populations of NR crystal structures without co-activator peptide (light grey) and with co-activator peptide (dark grey). Analysis of available NR structures reveals that D_1 between the charge clamp amino acids without peptide is very variable and ranges from 10 to 26 Å. In the structures bound to the peptide, the distance D_1 is much less variable ranging from 17 to 22 Å, only, indicating that the co-activator peptide "locks" certain conformations and therefore has substantial influence on the conformation of NRs. Source data are provided as a Source Data file.

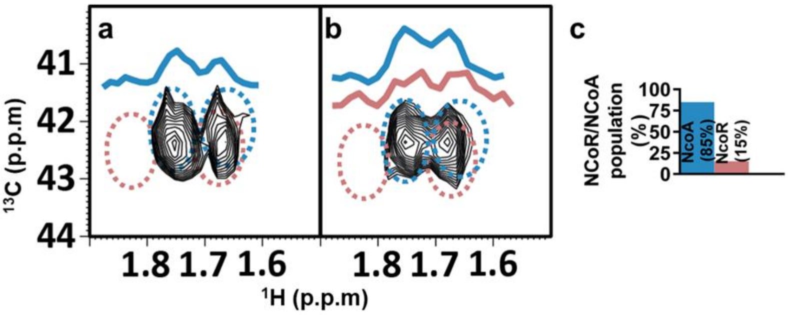

Supplementary Figure 7: Influence of antagonist (Guggulsterone) on the binding of co-regulator peptides to FXR. Monitoring of the NMR signal (Hβ of leucine in NCoA/NCoR) in response to addition of FXR, followed by addition of guggulsterone and the coactivator peptide NCoA. (a) Cβ of leucine in NCoA in the presence of guggulsterone and FXR. Strong presence of the signal clearly indicates that NCoA is only weakly recruited to the FXR-LBD. (b) Spectrum resulting upon addition of NCoR to (a). The repressor signals are markedly line-broadened due to binding to FXR in presence of the antagonist guggulsterone and the NCoA peptide is further released. (c) Graph representing the relative populations of the released NCoA/NCoR peptides upon binding of the ligand. Note: The experimental conditions were equal to Figure 5c except that the order of addition of the peptides was reversed (NCoA preceded the NCoR peptide).

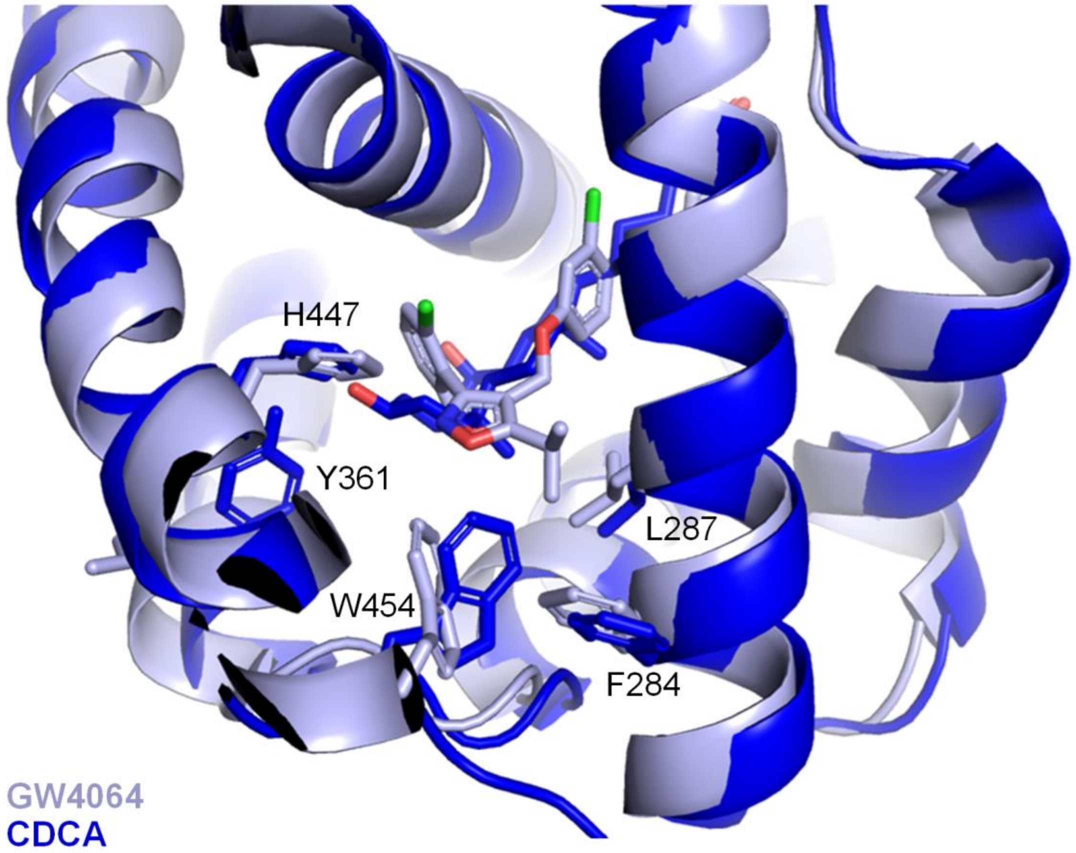

Supplementary Figure 8: Superimposed binding modes of GW4064 (light blue, 3DCT) and CDCA (dark blue, 6HL1) in the FXR-LBD. W454 seems to be involved in binding of CDCA but not GW4064 which agrees with the observation that W454 mutation affects the activity of CDCA but not of GW4064.

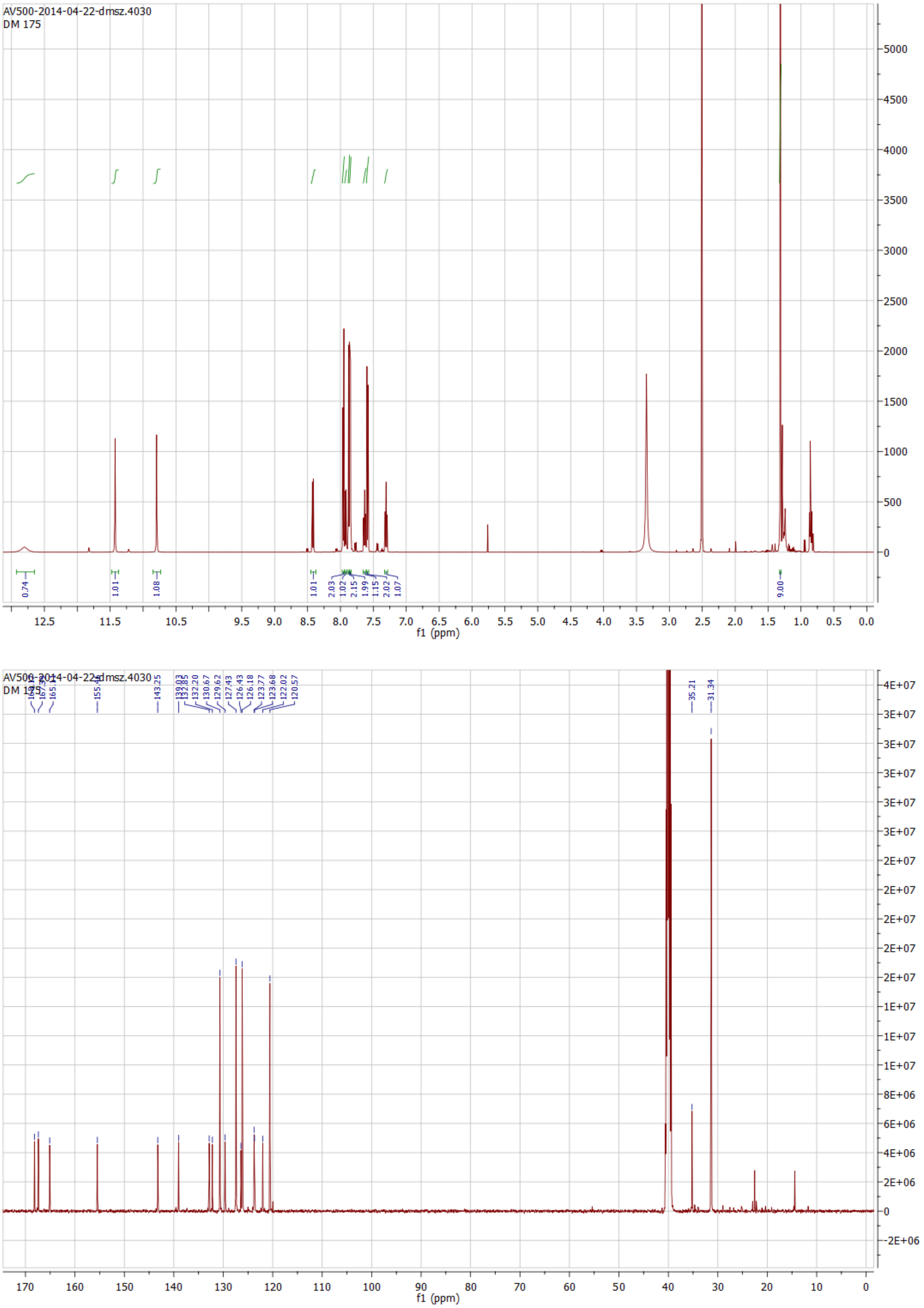

Supplementary Figure 9: ^{1}H- and ^{13}C-NMR of partial agonist 1.

Supplementary Tables

Supplementary Table 1: Crystal structure data collection and refinement statistics.

	Apo	CDCA	DM175
Beam line	BESSY BL14.2	BESSY BL14.2	BESSY BL14.2
**Data collection**			
Resolution (Å) [a]	41.58-1.66 (1.75-1.66)	34.24 -1.60 (1.70-1.60)	80.43-2.61 (3.70-2.61)
Space group	C2	C2	P1
Cell dimensions			
a, b, c (Å)	85.36 34.56 82.88	84.44 33.82 83.04	34.48 48.68 82.20
α, β, γ (°)	90.00 103.05 90.00	90.00 101.47 90.00	88.89 78.10 85.07
R_{meas} [b] (%)	14.0 (367.7)	4.9 (84.6)	16.1 (34.1)
Wavelength (Å)	0.91841	0.98004	0.91841
Mean I / σ(I)	6.8 (0.3)	24.1 (1.8)	9.6 (5.0)
Completeness (%)	95.5 (74.0)	95.4 (75.4)	98.9 (98.7)
No. of unique reflections	26862 (2941)	29422 (3739)	15595
Multiplicity	3.6 (2.7)	6.7 (4.6)	4.0 (4.0)
CC(1/2)	0.996 (0.123)	0.999 (0.736)	0.993 (0.953)
**Refinement**			
R_{work} [c]	0.2153	0.2010	0.2591
R_{free} [d]	0.2458	0.2461	0.2901
B-factors (overall)	35.1	36.9	47.0
Protein	34.8	36.4	46.7
Ligand	-	27.3	80.7
Water	40.3	43.2	30.8
Rms bonds (Å)	0.007	0.010	0.025
Rms angles (°)	0.898	1.361	1.297
**Ramachandran Plot**			
Favored (%)	97.51	97.44	95.3
Allowed (%)	2.49	2.56	4.7
PDB code	6HL0	6HL1	4QE8

[a] Values in parentheses are for highest-resolution shell.

[b] $R_{meas} = \Sigma_{hkl} (N/(N-1))^{1/2} \Sigma_i | I_i (hkl) - < I (hkl) > | / \Sigma_{hkl} \Sigma_i I_i (hkl)$, where N is the number of observations of the reflection with index hkl and I_i is the intensity of its ith observation.

[c] $R_{work} = |F_{obs} - F_{calc}| / |F_{obs}|$, where F_{obs} and F_{calc} are the observed and calculated structure factors, respectively.

[d] R_{free} is calculated as R_{work} using 5% of all reflections randomly chosen and excluded from structure calculation and refinement.

Supplementary Table 2: Primers used for qRT-PCR experiments. From[1–3].

	forward primer	reverse primer
SHP (human)	5`-GCTGTCTGGAGTCCTTCTGG	5`-CCAATGATAGGGCGAAAGAAGAG
CYP7A1 (human)	5`-CACCTTGAGGACGGTTCCTA	5`-CGATCCAAAGGGCATGTAGT
BSEP (human)	5`-CATGGTGCAAGAAGTGCTGAGT	5`-AAGCGATGAGCAACTGAAATGAT
OSTα (human)	5`-TGCTGCTCACCAGGAAGAAG	5`-ATAGAGCTGTGCTCCCCTCA
IBABP (human)	5`-TCAAGGCCACTGTGCAGATG	5`-CAGCTTGTCACCCACGATCTC
FGF19 (human)	5`-GGCCACTTGGAATCTGACATG	5`-TCCGGTGACAAGCCCAAAT
GAPDH (human)	5`-ATATGATTCCACCCATGGCA	5` GATGATGACCCTTTTGGCTC
SHP (mouse)	5`- GCAGGTCGTCCGACTATTCTGTAT	5`- GCAGTGGCTGTGAGATGCA
CYP7A1 (mouse)	5`- CTCCAGGGAGATGCTCTGTGTT	5`- GGTCTCATGACAGATTGGAGGTT
GAPDH (mouse)	5`- ACCACAGTCCATGCCATCAC[1]	5`- CACCACCCTGTTGCTGTAGCC

Supplementary Table 3: Key pharmacokinetic parameters of partial agonist 1.

C_{max} [ng/mL]	t_{max} [h]	$t_{1/2}$ [h]	t_z [h]	V_z/f [l/kg]	CL/f [l/(h*kg)]
1443	1.0	2.1	8.0	8177.4	2743.8

Supplementary Methods

Determination of aqueous solubility: 3 mg of compound **1** and 2 mL H2O dest. were inserted into a Whatman Uniprep filter (Whatman plc, Maidstone, UK) vessel and the mixture was shaken at 37°C for 24 h. The mixture was then pressed through the Uniprep filter and the concentration of dissolved compound **1** in filtrate was quantified by HPLC (Waters 600 Controller and Waters 2487 Dual Absorbance Detector equipped with a MultoHigh100 Phenyl 5 µ 240+4 mm column, CS-Chromatographie Service GmbH) using external calibration.

Determination of lipophilicity: The logP value of compound **1** was determined by HPLC analysis using a VWR Hitachi Chromaster System with DAD 5430. The HPLC column was a MultoHigh 100RP18 (4,6 mm I.D., 250mm length, 5 µ particle size) from Chromatographie-Service GmbH (Langerwehe, Germany). A linear gradient was used with mobile phase A as 100% acetonitrile, and mobile phase B as 100% 10 mM ammonium acetate (adjusted to pH 7.4 with ammonium hydroxide and acetic acid). The gradient table was: 0 min/ 5% A, 2.0 min/ 5% A, 12.0 min/ 95% A, 20 min/ 95% A, 30 min/ 5% A, 35 min/ 5% A. Flow rate was 0.5 ml/min, and UV spectra were collected at 254 nm and 280 nm. The samples were dissolved in DMSO at 30 µM and 50 µL were injected.
The HPLC capacity factor k' was determined according to $k' = (t_R - t_0)/t_0$. Where t_R is the retention time and t_0 the retention time of the unretained reference compound (thiourea). The logP was calibrated to k' by running 11 reference compounds (see Source Data file) and plotting k' versus literature logP values[4].

Supplementary references

1. Park, S. W. *et al.* Cytokines induce small intestine and liver injury after renal ischemia or nephrectomy. *Lab. Invest.* **91**, 63–84 (2011).
2. Merk, D. *et al.* Extending the structure-activity relationship of anthranilic acid derivatives as farnesoid X receptor modulators: development of a highly potent partial farnesoid X receptor agonist. *J. Med. Chem.* **57**, 8035–8055 (2014).
3. Schmidt, J. *et al.* A dual modulator of farnesoid X receptor and soluble epoxide hydrolase to counter nonalcoholic steatohepatitis. *J. Med. Chem.* **60**, 7703–7724 (2017).
4. Kim, S. *et al.* PubChem Substance and Compound databases. *Nucleic Acids Res.* **44**, D1202–D1213 (2016).
