# Supplementary material for: Molecular tuning of farnesoid X receptor partial agonism
Source: Nat Commun. 2019 Jul 2;10:2915. doi: 10.1038/s41467-019-10853-2 (PMC6606567; doi:10.1038/s41467-019-10853-2)
Supplement: Supplementary file 3 — Reporting Summary [file 41467_2019_10853_MOESM3_ESM.pdf]

nature research

Corresponding author(s): Daniel Merk, Harald Schwalbe

Last updated by author(s): May 20, 2019

Reporting Summary

Nature Research wishes to improve the reproducibility of the work that we publish. This form provides structure for consistency and transparency in reporting. For further information on Nature Research policies, see Authors & Referees and the Editorial Policy Checklist.

Statistics

For all statistical analyses, confirm that the following items are present in the figure legend, table legend, main text, or Methods section.

n/a	Confirmed	
☐	☒	The exact sample size (*n*) for each experimental group/condition, given as a discrete number and unit of measurement
☐	☒	A statement on whether measurements were taken from distinct samples or whether the same sample was measured repeatedly
☐	☒	The statistical test(s) used AND whether they are one- or two-sided<br>*Only common tests should be described solely by name; describe more complex techniques in the Methods section.*
☒	☐	A description of all covariates tested
☒	☐	A description of any assumptions or corrections, such as tests of normality and adjustment for multiple comparisons
☒	☐	A full description of the statistical parameters including central tendency (e.g. means) or other basic estimates (e.g. regression coefficient) AND variation (e.g. standard deviation) or associated estimates of uncertainty (e.g. confidence intervals)
☐	☒	For null hypothesis testing, the test statistic (e.g. *F*, *t*, *r*) with confidence intervals, effect sizes, degrees of freedom and *P* value noted<br>*Give P values as exact values whenever suitable.*
☒	☐	For Bayesian analysis, information on the choice of priors and Markov chain Monte Carlo settings
☒	☐	For hierarchical and complex designs, identification of the appropriate level for tests and full reporting of outcomes
☒	☐	Estimates of effect sizes (e.g. Cohen's *d*, Pearson's *r*), indicating how they were calculated

Our web collection on statistics for biologists contains articles on many of the points above.

Software and code

Policy information about availability of computer code

Data collection	NMR spectra were recorded using Bruker software. X-ray data was collected at BESSY BL14.2.
Data analysis	Pharmacological data was analysed using Microsoft Excel, GraphPad Prsim 7 and Kinetica 5.0. NMR data was analysed using Bruker software. X-ray data was processed and refined using autoPROC, XDSAPP and PHENIX.

For manuscripts utilizing custom algorithms or software that are central to the research but not yet described in published literature, software must be made available to editors/reviewers. We strongly encourage code deposition in a community repository (e.g. GitHub). See the Nature Research guidelines for submitting code & software for further information.

Data

Policy information about availability of data

All manuscripts must include a data availability statement. This statement should provide the following information, where applicable:

- Accession codes, unique identifiers, or web links for publicly available datasets
- A list of figures that have associated raw data
- A description of any restrictions on data availability

Macromolecular data has been deposited in the PDB with the accession codes 4QE8, 6HL0, and 6HL1. The source data underlying Figure 1, Table 1, Supplementary Figure 4 and Supplementary Figure 6 are provided as a Source Data file. Other data are available from the corresponding authors on reasonable request.

Field-specific reporting

Please select the one below that is the best fit for your research. If you are not sure, read the appropriate sections before making your selection.

☒ Life sciences ☐ Behavioural & social sciences ☐ Ecological, evolutionary & environmental sciences

For a reference copy of the document with all sections, see nature.com/documents/nr-reporting-summary-flat.pdf

Life sciences study design

All studies must disclose on these points even when the disclosure is negative.

Sample size	No sample size calculation was performed. Pharmacological/biochemical experiments were performed with common numbers of independent repeats (at least three independet biological samples).
Data exclusions	No data was exculded.
Replication	In vitro pharmacological experiments and the in vivo study used common techniques and all information for reproduction is given in the manuscript or Supplementary Information.
Randomization	Animals were randomized to receive the experimental drug or vehicle. For other aspects of the present study, randomization was not relevant.
Blinding	Animal samples were blinded. For other parts of the study, blinding was not relevant.

Reporting for specific materials, systems and methods

We require information from authors about some types of materials, experimental systems and methods used in many studies. Here, indicate whether each material, system or method listed is relevant to your study. If you are not sure if a list item applies to your research, read the appropriate section before selecting a response.

Materials & experimental systems

n/a	Involved in the study	
☒	☐	Antibodies
☐	☒	Eukaryotic cell lines
☒	☐	Palaeontology
☐	☒	Animals and other organisms
☒	☐	Human research participants
☒	☐	Clinical data

Methods

n/a	Involved in the study	
☒	☐	ChIP-seq
☒	☐	Flow cytometry
☒	☐	MRI-based neuroimaging

Eukaryotic cell lines

Policy information about cell lines

Cell line source(s)	HeLa cells: DSMZ, HepG2 cells: DSMZ, HEK293 cells: DSMZ
Authentication	None of the cell lines were authenticated.
Mycoplasma contamination	All cell lines were regularly tested for absence of mycoplasma contamination
Commonly misidentified lines (See ICLAC register)	n/a

Animals and other organisms

Policy information about studies involving animals; ARRIVE guidelines recommended for reporting animal research

Laboratory animals	The study involved male C57BL/6JRj mice.
Wild animals	The study involved no wild animals.
Field-collected samples	The study involved no field collected samples.

Ethics oversight	The study was approved by Landesamt für Gesundheit und Verbraucherschutz, Abteilung Lebensmittel- und Veterinärwesen, Saarbrücken.

Note that full information on the approval of the study protocol must also be provided in the manuscript.
